# The chromosome-scale reference genome and transcriptome analysis of *Solanum torvum* provides insights into resistance to root-knot nematodes

**DOI:** 10.3389/fpls.2023.1210513

**Published:** 2023-07-17

**Authors:** Hongyuan Zhang, Hao Chen, Jie Tan, Shuping Huang, Xia Chen, Hongxia Dong, Ru Zhang, Yikui Wang, Benqi Wang, Xueqiong Xiao, Zonglie Hong, Junhong Zhang, Jihong Hu, Min Zhang

**Affiliations:** ^1^ Institute of Vegetable Research, Wuhan Academy of Agricultural Sciences, Wuhan, Hubei, China; ^2^ State Key Laboratory of Crop Stress Biology for Arid Areas, College of Agronomy, Northwest A&F University, Yangling, Shaanxi, China; ^3^ Institute of Vegetable Research, Guangxi Academy of Agricultural Sciences, Nanning, Guangxi, China; ^4^ State Key Laboratory of Agricultural Microbiology, Huazhong Agricultural University, Wuhan, China; ^5^ Department of Plant Sciences, University of Idaho, Moscow, ID, United States; ^6^ National Key Laboratory for Germplasm Innovation & Utilization of Horticultural Crops, Huazhong Agricultural University, Wuhan, Hubei, China

**Keywords:** *Solanum torvum*, genome, transcriptome, root-knot nematode, ABC transporter

## Abstract

*Solanum torvum* (Swartz) (2n = 24) is a wild Solanaceae plant with high economic value that is used as a rootstock in grafting for Solanaceae plants to improve the resistance to a soil-borne disease caused by root-knot nematodes (RKNs). However, the lack of a high-quality reference genome of *S*. *torvum* hinders research on the genetic basis for disease resistance and application in horticulture. Herein, we present a chromosome-level assembly of genomic sequences for *S*. *torvum* combining PacBio long reads (HiFi reads), Illumina short reads and Hi-C scaffolding technology. The assembled genome size is ~1.25 Gb with a contig N50 and scaffold N50 of 38.65 Mb and 103.02 Mb, respectively as well as a BUSCO estimate of 98%. GO enrichment and KEGG pathway analysis of the unique *S*. *torvum* genes, including *NLR* and ABC transporters, revealed that they were involved in disease resistance processes. RNA-seq data also confirmed that 48 *NLR* genes were highly expressed in roots and fibrous roots and that three homologous *NLR* genes (*Sto0288260.1*, *Sto0201960.1* and *Sto0265490.1*) in *S. torvum* were significantly upregulated after RKN infection. Two ABC transporters, *ABCB9* and *ABCB11* were identified as the hub genes in response to RKN infection. The chromosome-scale reference genome of the *S*. *torvum* will provide insights into RKN resistance.

## Introduction

*Solanum torvum* (Swartz) (2n = 24) is a wild Solanaceae plant with high economic value, and its roots, leaves, stems and fruits can be used as medicine for the relief of swelling and pain (Govindaraju et al., 2010). *S. torvum*, commonly known as turkey berry, is distributed in most pantropical areas, including Pakistan, India, China, and tropical America. Eggplant (*Solanum melongena* L.) is a vegetable crop species belonging to the Solanaceae family, which includes economically important species such as tomato (*S. lycopersicum* L.), potato (*S. tuberosum* L.), pepper (*Capsicum annuum* L.), and tobacco (*Nicotiana tabacum* L.) (Hirakawa et al., 2014). Root-knot nematodes (RKNs, *Meloidogyne* spp.) are destructive endoparasites that mainly parasitize a variety of cultivated plants. In many countries, the production of eggplant is seriously threatened by RKNs. Once infected with RKNs, large knots form in the roots of the host plant. They eventually lead to giant cells that are multinucleated and hypertrophic cells that are a source of nutrients for nematodes to grow, so that the surrounding root tissue cannot obtain enough nutrients for them to grow (Ling et al., 2017). There is evidence that *S*. *torvum*, is resistant to serious soil borne diseases caused by bacteria, fungal wilt and RKNs (Bagnaresi et al., 2013; Zhou et al., 2018). Therefore, *S*. *torvum* is often used as rootstock in the grafting of solanaceous plants to improve the resistance to soil borne diseases caused by RKNs (Gousset et al., 2005).

The ban on highly toxic nematicides makes it urgent to need environmentally friendly and pesticide free control methods, and the breeding of RKNs resistant varieties seems to be a promising alternative. Therefore, the identification of nematode resistance (Nem-R) may be of great importance for eggplant cultivation. Using genome-specific satellite markers and chromosome breakpoint analysis, the *Hs1* (*pro-1*) gene with resistance to the beet cyst nematode (Heterodera schachtii Schmidt) was cloned from sugar beet (*Beta vulgaris* L.) (Cai et al., 1997). Nem-R genes cloned from tomato or potato relatives, including *Mi-1*, *Hero A*, *Gpa2* and *Gro1-4*, are very similar to known plant *R*-genes in their domains and belong to NBS-LRR like *R*-genes (Williamson and Kumar, 2006). With regard to the subclass of RKN *R*-genes (RKN-R), almost no resistance genes were found, and only *Mi-1* from tomato and *Ma* from Myrobalan plum were cloned. The *Mi-1* gene with resistance to RKN was cloned from tomato by bacterial artificial chromosome cloning and isolation. This gene encodes a protein sharing structural characteristics with leucine rich repetitive plant resistance genes (Milligan et al., 1998; Vos et al., 1998). The *Ma* gene of RKN resistance has full spectrum and heat stable resistance to Meloidogvne. In contrast to *Mi-I* of tomato, the latter has more limited spectrum and lower efficiency under high temperature (Claverie et al., 2004). Pepper resistance to RKNs is controlled by the *Me* gene, which is thought to play an independent role in gene interactions (Djian-Caporalino et al., 2001; Gisbert et al., 2013). To identify the defense mechanism of RKNs in Solanaceae, some studies compared the transcriptional characteristics of eggplant and *S*. *torvum*, and found that many plant hormone related genes and transcription factors, such as *MYB*, *WRKY* and *NAC*, were differentially expressed at different time points after infection with RKNs (Zhang et al., 2021).

*NLR* (nucleotide-binding and leucine rich repeat) gene belongs to one of the largest gene families in plants. Most of the plant resistance genes (*R*-genes) are characterized by molecules belonging to the NLR receptor family, and are prone to duplication and translocation, with high sequence diversity (Chovelon et al., 2021). Most NLRs share a common biological structural basis, including a highly conserved nucleic acid binding domain at the N-terminal (nucleotide binding, NB) and a highly variable leucine rich repeat at the C-terminal (leucine rich repeat, LRR) (Zheng et al., 2022). These large and abundant proteins are mainly involved in the detection of various pathogens and many *NLR* genes only show activated expression under pathogen invasion, although some *NLR* members still show tissue/space specific expression in wild type plants (McHale et al., 2006). In wild eggplant *Solanum aculeatissimum*, a nucleotide binding site rich repeat (NBS-LRR) resistance gene, *SacMi* was isolated and characterized. Silencing of *SacMi* enhanced the susceptibility of *S. aculatissimum* to RKNs, indicating that *SacMi* may participate in the resistance to RKNs (Zhou et al., 2018).

Although it is very important for other solanaceous crops, especially eggplant, to identify the resistance of *S*. *torvum* to RKN, there is little research on the resistance mechanism of *S*. *torvum* at present. The main reason for this phenomenon is the lack of high-quality *S*. *torvum* genome. Combined genomic and transcriptome analysis could elucidate the possible mechanism underlying the RKN disease resistance (Hu et al., 2022; Zhang et al., 2022; Zhao et al., 2022). Therefore, it is necessary to obtain a high-quality chromosome-scale genome of *S. torvum* and study the mechanism for RKN resistance of *S. torvum* at the whole-genome level.

In this study, we sequenced and assembled the whole genome of *S*. *torvum* using Illumina paired-end reads, PacBio sequencing, and Hi-C technology. We structurally and functionally annotated the genome with clustered gene families related to diverse biological processes. Our work provides a valuable resource for revealing the evolution of *S*. *torvum* and for understanding the mechanism of resistance to RKNs.

## Materials and methods

### Plant materials

*Solanum torvum* (Swartz) was planted in the field of the Wuhan Vegetable Research Institute, Wuhan Academy of Agricultural Sciences (30.82°N, 114.37°E), Wuhan, China. The tender leaves at the seedling stage were collected for further experiments. Leaves, roots, stems, fibrous roots, buds, flowers and fruits were harvested at the corresponding stage, immediately frozen in liquid nitrogen, and stored at –80°C until use.

### Library construction and genome sequencing

High-quality genomic DNA was extracted from tender leaves of *S*. *torvum* using a Qiagen DNeasy Plant Mini Kit. For third-generation genome sequencing, the SMRT Bell library was prepared using the SMRTbell Express Template Prep Kit 2.0, which was performed using SMRT sequencing on a PacBio Sequel system following the manufacturer’s standard protocol. To estimate genome size, the Illumina DNA libraries were constructed using the Next Ultra DNA library prep kit (NEB) and sequenced on the HiSeq X Ten platform (Illumina, San Diego, USA).

### Genome assembly and assessment

Before genome assembly, the read information obtained by sequencing to estimate genome features was subjected to *17*-mer analysis to estimate the genome size. We assembled the *S*. *torvum* genome based on PacBio subreads, Illumina short reads, and Hi-C data using a hierarchical method. The longer PacBio subreads were *de novo* assembled by Canu v1.5 (Koren et al., 2017). All the PacBio reads were then mapped to the previously assembled contigs using minimap2 (Li, 2016) and further corrected with arrow software. To increase the accuracy of assembly, all the filtered Illumina reads were mapped to the corrected contigs with BWA-mem (Li, 2013) and further corrected by Pilon (Walker et al., 2014) to correct indel errors associated with homopolymer repeats in the PacBio data. Hi-C is a technology derived from chromosome conformation capture technology that utilizes high-throughput data and is mainly used to assist in genome assembly. To anchor scaffolds onto pseudochromosomes, HiCUP v0.6.1 (Wingett et al., 2015) was used to map and process the reads from the Hi-C library. For Hi-C analysis, the raw reads were trimmed to obtain clean Hi-C reads. Then, the obtained clean reads were compared with the preassembled contigs using Juicer (Durand et al., 2016). After filtering the results and removing the misaligned reads, 3D-DNA software was used to preliminarily cluster, sequence, and direct the pseudochromosomes (Dudchenko et al., 2017). Juicer-box was used to adjust, reset, and cluster the pseudochromosomes to improve the chromosome assembly quality. For the evaluation of the Hi-C assembly results, the final pseudochromosome assemblies were divided into 500 kb bins of equal lengths, and a heatmap was used to visualize the interaction signals generated by the valid mapped read pairs between each bin.

To assess genome assembly quality, the Benchmarking Universal Single-Copy Orthologs v3 (BUSCO) tool (http://busco.ezlab.org/) (Simão et al., 2015) was used with single-copy orthologous genes. Genome integrity is assessed using Long Terminal Repeat through LTR_FINDER and LTR_retriever software, which is finally represented by the LAI index.

### Gene prediction and functional annotation

Repetitive sequences are an important part of a genome and are divided into two types, namely, tandem repeats and interspersed repeats. Two methods, *do novo* prediction and homology-based search, were used to annotate repeat sequences in the genome. Tandem repeats in genome sequences were found by TRF (Benson, 1999). RepeatMasker v4.1.3 (Tarailo-Graovac and Chen, 2009) and RepeatProteinMask v4.1.3 (http://www.repeatmasker.org) were used to identify repetitive sequences based on the Repbase database (Jurka et al., 2005). For *de novo* prediction, the final sequence file obtained by RepeatModeler v2.0.3 and LTR-FINDER (Xu and Wang, 2007) was used as the library, and the genome sequence was annotated through RepeatMaker software to obtain repetitive sequences. Finally, after integrating the above methods and removing redundancy, it will be used as the result of the last repetitive sequences.

The annotation of high-quality protein-coding genes was carried out by integrating *de novo*, homology-based and transcriptome-based predictions. AUGUSTUS v2.4 (Nachtweide and Stanke, 2019) and GlimmerHMM v3.04 (Majoros et al., 2004) was used for ab initio prediction. For homology-based prediction, protein sequences from six species (*S*. *torvum*, *S.pennellii*, *S.tuberosum*, *S.lycopersicum*, *C.annuum*, *N.sylvestris*) were analyzed using tblastn with a cutoffe-value of 1e-5, and aligned against the contents of Swiss-Prot, TrEMBL (http://www.UniProt.org/), NR and so on. The RNA-seq data of *S*. *torvum* were mapped to genome sequences through HISAT2 v2.0.4 (Kim et al., 2015) and StringTie v2.0.4 (Pertea et al., 2015). Finally, the MAKER package v2.31.878 (Cantarel et al., 2008) was used to annotate and integrate the results generated by the above methods.

For non-coding RNAs annotations, tRNAscan-SE (Lowe and Eddy, 1997) was used to annotate transfer RNA (tRNA) sequences. BLASTN (Kent, 2002) was used to search for ribosomal RNAs (rRNAs) and microRNAs (miRNAs), and snRNA sequences were predicted by Infernal 1.1 (http://eddylab.org/infernal/) against the Rfam database (Gardner et al., 2011) using default parameters.

### Phylogenetic and whole-genome duplication analyses

Homological relationships among the protein sequences derived from 10 plants (*S.torvum*, *S.melongena*, *S.tuberosum*, *S.aethiopicum*, *C.annuum*, *S.lycopersicum*, *A.thaliana*, *N.tabacum*, *P.inflata*, *O.sativa*) were determined by BLAST analysis. The distribution of orthologous gene families from the 10 different species was identified using the OrthoMCL package v2.0.9 (Li et al., 2003). After gene family clustering, we aligned all high-quality single-copy orthologous gene protein sequences using MUSCLE v3.8.31 (Edgar, 2004) and constructed a phylogenetic tree using PhyML v3.1 (http://www.atgc-montpellier.fr/phyml/versions.php). CAFE v4.2 (Han et al., 2013) was used to calculate the number of gene families amplified and contracted on each phylogenetic tree branch based on divergence time and gene family clustering.

We used the synonymous substitution rate (*K*s) to infer the occurrence of WGD events. First, BLASTP was used to search for putative paralogous and orthologous genes by aligning the genomes of *S. lycopersicum*, *S. torvum*, and *S. melongena*. Then, syntenic blocks within and among species were identified using MCScanX (Wang et al., 2012). Subsequently, *K*s values of each gene pair were calculated with KaKs_Calculator 2.0 (Wang et al., 2010), and four-fold synonymous third-codon transversion (4DTv) rate was determined using a Perl script (https://github.com/JinfengChen/Scripts). The WGD events in the *S*. *torvum* genome were determined by plotting the distribution frequency of the 4DTv values.

### Transcriptome sequencing

Total RNA was isolated from different tissues (leaf, stem, bud, flower, fruit, fibrous root and root) of *S. torvum* (Swartz) with three biological replicates using TRIzol reagent (Tiangen, Beijing, China). Approximately 2 μg of high-quality RNA per sample was used for sequencing library construction as previous described (Zhang et al., 2021). The 150 bp paired-end sequencing was performed on the Illumina HiSeq 4000 platform (Illumina, San Diego, USA). The transcriptome sequencing data were also used for genomic gene prediction and annotation. We used fastp (Chen et al., 2018) to remove adaptors from raw reads and filter out low-quality reads. Clean reads were compared to our assembled *S.torvum* genome using HISAT2, and transcripts were analyzed using Stringtie v2.0.4. After comparing the existing annotations with cuffcompare v2.2.1, new transcripts were obtained, and new transcripts with protein coding potential confirmed by CPC2 (Kang et al., 2017) were added to the gene set. Finally, the gene set was quantified through RSEM. The differentially expressed genes (DEGs) were identified with the DESeq2 R package v3.11 (Love et al., 2014) based on |log2 (fold-change)|≥1 and adjusted *P* value (*P* < 0.05).

### Co-expression network construction

A weighted gene co-expression network of differentially expressed genes was constructed using the WGCNA package in R (Langfelder and Horvath, 2008). An unsupervised co-expression relationship was built based on the adjacency matrix which represents the network connection strength between gene pairs. The one-step network construction option with a soft-thresholding power value of 10, min module size = 30 and merge cut height = 0.25 were used. The other parameters were set to default values. Highly similar modules were subsequently identified by clustering and then merged together to new modules on the bias of eigengenes. The correlation of each module was also analyzed and visualized by a heatmap. Then, the co-expression network was visualized by Cytoscape software (Smoot et al., 2011).

## Results

### Genome sequencing and assembly

**Figure 1:**
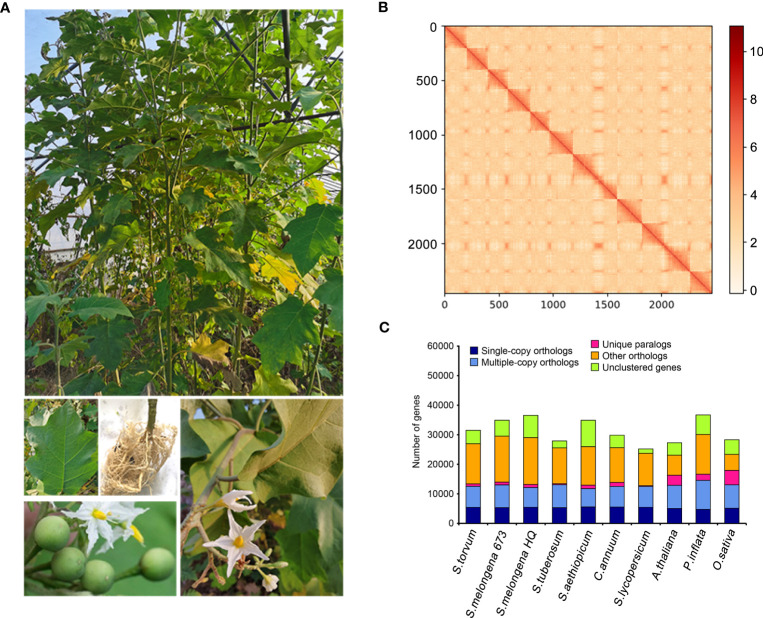
**(A)** Morphological characteristics of *S*. *torvum*. **(B)** Interchromosomal Hi-C contact map of *S*. *torvum*. **(C)** The distribution of orthologous genes in different species.

*Solanum torvum* is a shrub and its roots can resist to RKNs (**Figure 1A**).Using *k*-mer analysis (*k* = 17), we estimated that the *S*. *torvum* genome size is approximately 1, 185 Mb (**Supplementary Figure S1**). A total of 148.60 Gb of PacBio single-molecule long reads and 118.95 Gb of Illumina paired-end clean reads were generated for initial assembly. The original sketch length of the *S*. *torvum* genome sequence is 1.25 Gb with 309 contigs. To anchor the scaffolds to chromosomes, we constructed high-throughput chromosome conformation capture (Hi-C) libraries of *S. torvum*, generating 136.27 Gb clean Hi-C reads (**Figure 1B**). After Hi-C assisted genome assembly, the genome sequence and direction of *S*. *torvum* were finally determined. The genome was assembled, and approximately 98.80% of the contigs were anchored into the 12 pseudo-chromosomes, generating chromosome-level sequences of 1.25 Gb with a contig N50 and scaffold N50 of 38.65 Mb and 103.02 Mb, respectively, which significant higher than those of eggplant (**Table 1**; **Supplementary Tables 1**, **S2**).

**Table 1:** Global statistics of the *Solanum torvum* genome assembly and annotation compared with *Solanum melongena HQ*.

Assemebly & annotation	*S. torvum*	*S. melongena HQ*
Total length of assemblies (Gb)	1.25	1.07
No. of scaffolds	12	12
N50 of scaffolds (Mb)	103.02	89.64
No. of contigs	309	2263
Total length of contigs (Mb)	1,245.54	–
N50 of contigs (Mb)	38.65	5.26
Longest contig (Mb)	99.09	–
Complete BUSCOs (%)	98	94.2
Gene number	31,496	36,582
Transposable element (TE) (%)	76.73	69.08
Long terminal repeat (LTR) (%)	68.35	65.8
GC content (%)	36.6	35.94

**Figure 2:**
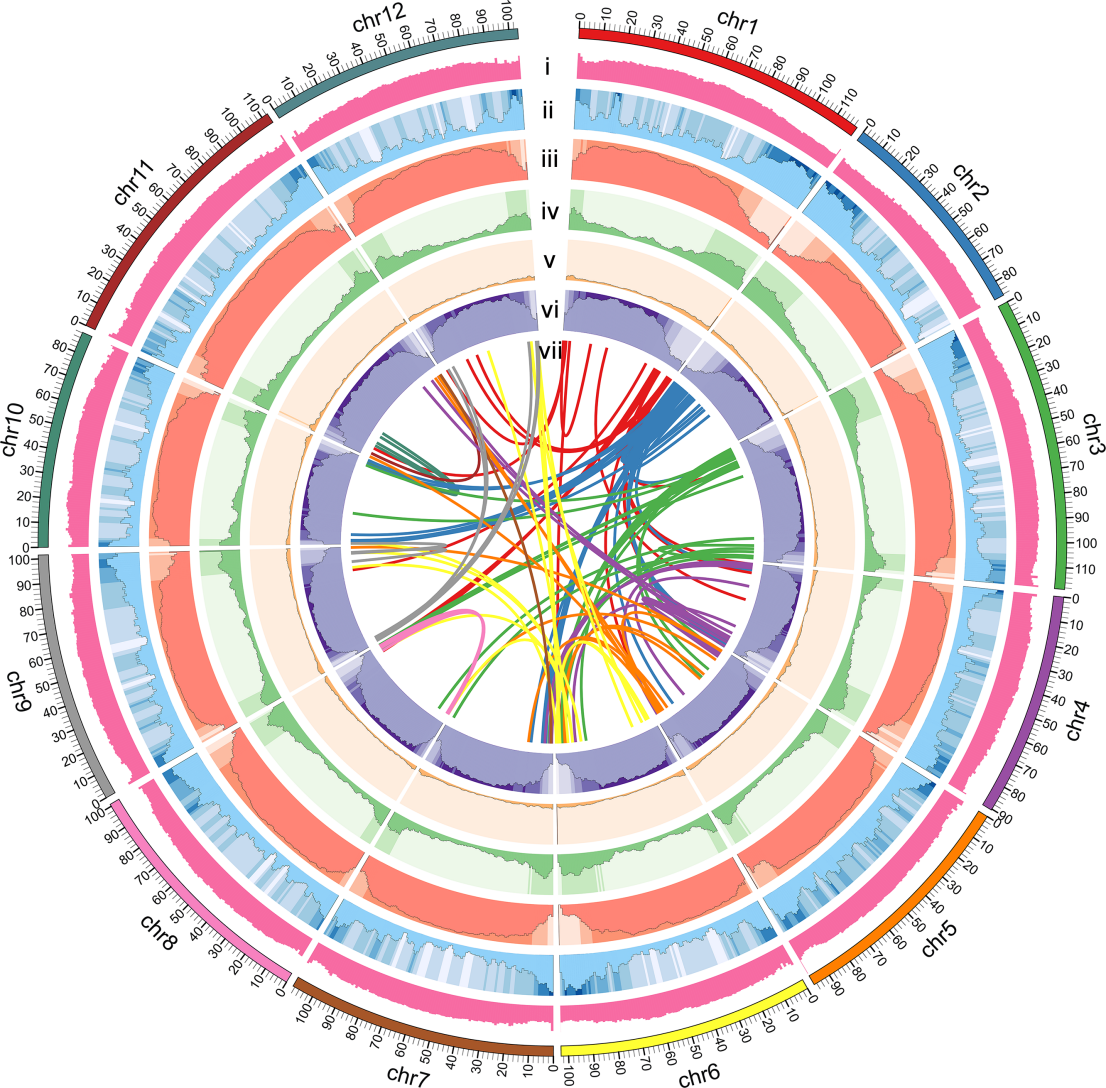
Circular map of *S*. *torvum* genome. (i) GC content, (ii) gene density, (iii) repeat content, (iv) DNA Transposon, (v) LINE, (vi) LTR content, and (vii) synteny relations.

BUSCO analysis showed that the completeness of the *S*. *torvum* genome was 98% with low redundancy (3.3% complete and duplicated BUSCOs) and the long terminal repeat (LTR) assembly index (LAI) had a high score of 10.47 (**Supplementary Table S3**). Moreover, to evaluate the sequence consistency, the software BWA was used to compare the Illumina paired-end clean reads to the assembled genome, and the paired mapping rate and coverage reached 98.60% and 99.68%, respectively. In addition, 17,714 gene families were constructed with 517 single-copy genes identified among the 11 species (**Figure 1C**). Therefore, these results indicated that the genome assembly is of high quality with high coverage (**Figure 2**).

### Repeat elements and gene annotation

The repetitive sequences of the *S*. *torvum* genome were annotated by combining *de novo* prediction and homology-based searches at the DNA and protein levels, identifying 955.75 Mb of repeated sequences accounting for 76.73% of the assembled genome, which is slightly higher than that of eggplant (Li et al., 2021) (**Supplementary Figure S2** and **Table S4**). Transposable elements (TEs) are the largest single component of most eukaryotic genetic materials, and play an important role in shaping eukaryotic genomes and promoting their evolution (Feschotte et al., 2002). In *S*. *torvum*, TEs accounted for 76.73% of the genome size, with long-terminal repeat (LTR) retrotransposons accounting for 68.35% of the genome as the most common type of transposon elements (**Figure 2** and **Supplementary Table S4**). As another major class of transposable elements, DNA transposons account for 5.17% (**Supplementary Figure S2** and **Table S4**). The proportions of TEs and LTRs were slightly higher than those in eggplant (**Table 1**) (Wei et al., 2020; Li et al., 2021).

A total of 31,496 high-quality protein-coding genes were annotated in the *S*. *torvum* genome, of which 27,719 (88.01%) were further detected by RNA sequencing (RNA-seq) in seven tissues (**Supplementary Tables S5**, **S6**). The number of protein-coding genes was comparable with that of eggplant (36,582 genes), tomato (35,768 genes), potato (39,028 genes) and pepper (34,903 genes), indicating that the number of genes in these related species is similar (Xu et al., 2011; Sato et al., 2012; Kim et al., 2014; Wei et al., 2020) (**Table 1**).

Comparative analysis of the genomes among the five (*S. aethiopicum*, *S. torvum*, *S. melongena*, *S. melongena* and *S. tuberosum*) and four (*S*. *torvum*, *S. melongena*, *S. melongena* and *S. tuberosum*) *Solanum* species identified a total of 12,856 and 13,958 gene orthologs, respectively (**Supplementary Figure S3**). We further assessed the quality of these annotated genes by comparing them with several closely related species (*S. pennellii*, *S. tuberosum*, *S. lycopersicum*, *C. annuum* and *N. sylvestris*) (**Supplementary Figure S4**). The accuracy of these predicted genes assessed by comparing gene features including the distribution of mRNA length, CDS length, exon length, intron length and the exon number, showed similar distribution patterns, revealing high confidence gene models (**Supplementary Figure S4**). Analysis of gene functional annotations using the InterPro, GO, KEGG, SwissProt, TrEMBL, and NR databases revealed that most of the genes of *S*. *torvum* have close homologs from other organisms in the public databases (**Supplementary Table S6**). Noncoding RNAs of *S*. *torvum*, including 192 microRNAs and 1561 tRNAs, were also identified (**Supplementary Table S7**).

### Evolution of the *S*. *torvum* genome

**Figure 3:**
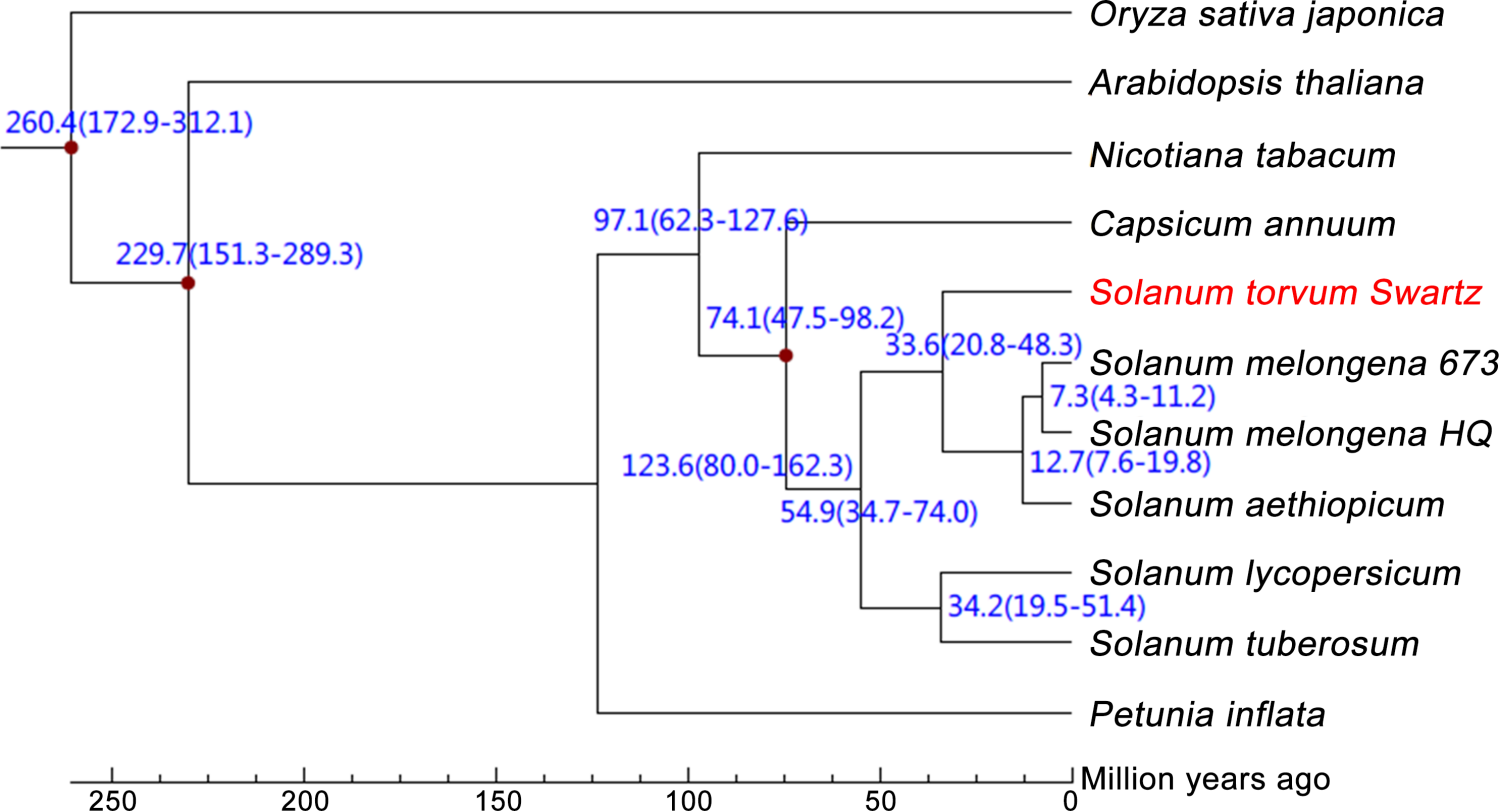
Phylogenetic tree showing divergence times based on ten sequenced plant genomes with *O. sativa* as an outgroup.

**Figure 4:**
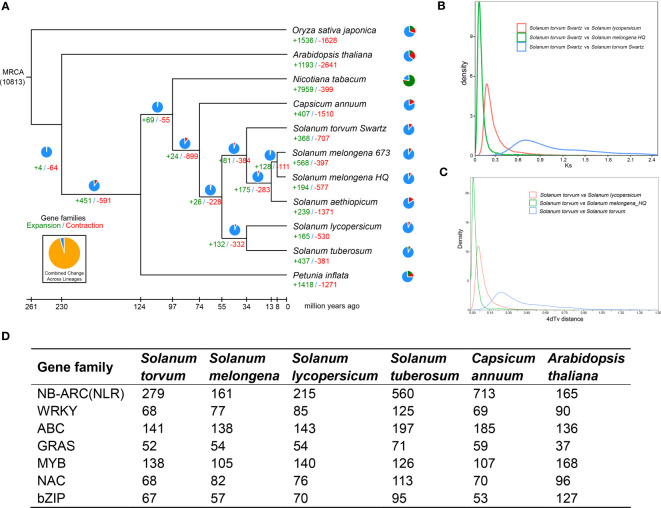
**(A)** Phylogenetic tree showing divergence times and the evolution of gene families in *S*. *torvum*. **(B)** Density distribution of Ks for paralogous gene pairs of the three Solanaceae genomes. **(C)** Age distribution of 4dTv values for the homologous gene pairs between the Solanaceae genomes. **(D)** Comparison of the number of different gene families between *S*. *torvum* and other species.

The expanded and contracted gene families of *S. torvum* were compared with those of nine other plant species with *O. sativa* as an outgroup. Phylogenetic analysis showed that *S*. *torvum* diverged from the common ancestor of *S*. *torvum*, *S*. *melongena* and *S. aethiopicum* for 33.6 million years ago (Mya), with a confidence interval ranging from 20.8 to 48.3 Mya (**Figure 3**). Moreover, 368 gene families in *S*. *torvum* underwent expansion, including the ATP-binding cassette (ABC) transporter and nucleotide-binding leucine-rich repeat (*NLR*) gene families, while 707 gene families were contracted (**Figure 4A**). GO enrichment analysis of these unique *S*. *torvum* gene families showed that they participate in a series of biological processes, such as purine nucleotide metabolic processes and chitin metabolic processes, which may explain why they are resistant to RKNs (Zhou et al., 2020). KEGG pathway analysis revealed that these expanded genes were involved in plant-pathogen interaction, diterpenoid biosynthesis, monoterpenoid biosynthesis and phenylpropanoid biosynthesis (**Supplementary Table S8**). These pathways were reported in response to pathogen infection, suggesting their possible roles in the resistance to RKNs in *S*. *torvum* (Kyndt et al., 2012). Comparative genome analysis revealed that 26 genes were positively selected in the *S*. *torvum* genome (**Supplementary Table S9**).

### Synteny analysis and whole-genome duplication

**Figure 5:**
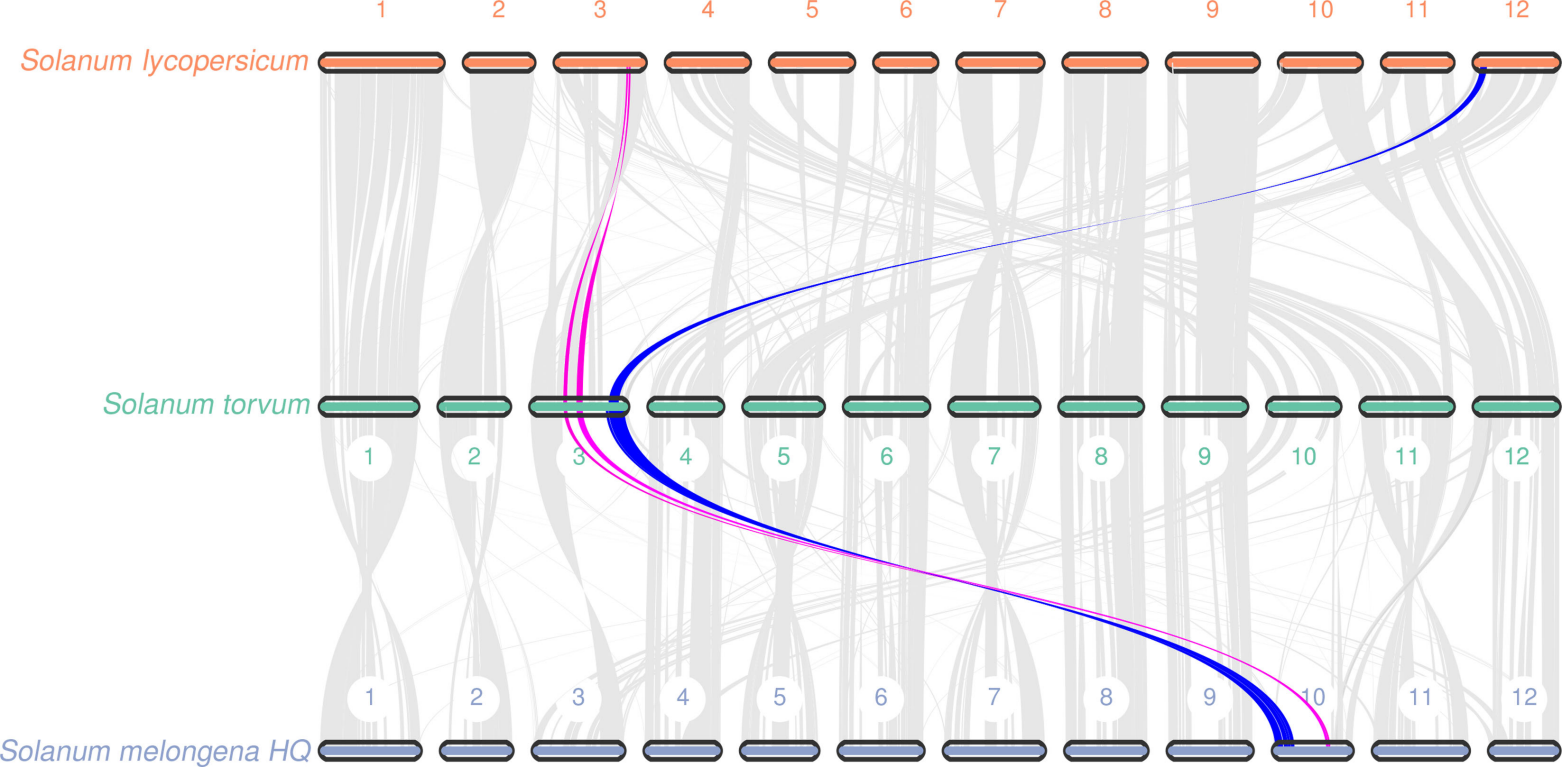
Collinear relationship at the chromosome level among *S*. *torvum*, *S*. *lycopersicum* and *S*. *melongena*. The red and blue lines showed the collinearity of genomic sequences on the chromosome 3 with *S*. *lycopersicum* and *S*. *melongena*.

Gene replication provides evolutionary potential for generating new functions. WGD doubles are strongly supported by the evidence common in many species-rich lineages of eukaryotes, so they are considered as the main driving force of species diversity (Song et al., 2012; Ren et al., 2018). To determine the occurrence of WGD events in *S*. *torvum*, the distributions of synonymous substitution rates (*K*s) and distance-transversion rates (4DTv) of paralogous gene pairs were estimated to shape a WGD event in *S*. *torvum* (**Figures 4B, C**). The divergence of these three species is consistent with the phylogenetic analysis (**Figure 4A**), confirming the accuracy of the present results (Li et al., 2021). Comparison of the gene families among different species in Solanaceae revealed some genes were expanded in *S. torvum* (**Figure 4D**). Population history analysis showed that the effective population size of *S*. *torvum* expanded along with that of *S*. *melongena* and then declined to a stable level, suggesting a possible concerted evolution (**Supplementary Figure S5**). Genome collinearity analysis of the three *Solanum* species indicated that some chromosomes were conserved, but chromosomes 3, 4, 5 and chromosomes 10, 11, 12 contained a large percentage of collinear regions among *S*. *lycopersicum*, *S*. *torvum* and *S*. *melongena* (**Figure 5**).

### *S*. *torvum* resistance to RKNs

**Figure 6:**
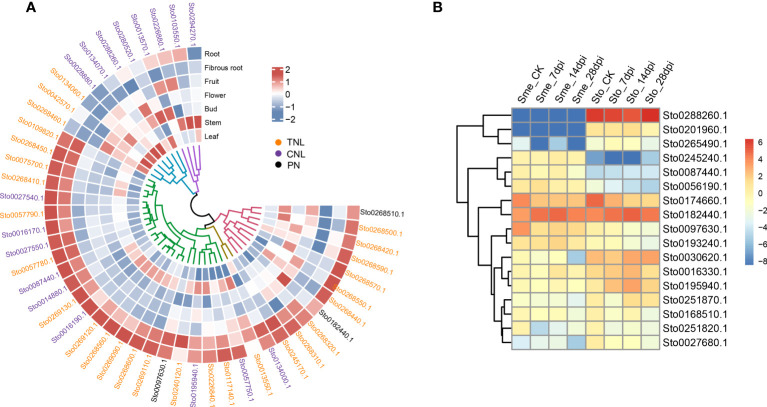
**(A)** Heatmap showing the expression patterns of 48 *NLR* genes. Expression levels with log2(FPKM). **(B)** The expression patterns of *NLR* genes in *S*. *torvum* (Sto) and their homologous genes in *S*. *melongena* (Sme) in response to root-knot nematodes (RKNs).

Many RKN resistance genes in tomato, pepper and other plants have been identified to as *NLR* genes containing NB-ARC domains (Milligan et al., 1998). In this study, we detected dramatic expansions of 279 *NLR* genes within 26 gene clusters and 68 WRKY transcription factors, compared with those in *S*. *melongena* (**Figure 4D**; **Supplementary Figures S6**-**S9** and **Tables S10**, **S11**), suggesting their roles in disease resistance. The *NLR* gene was mainly located at both ends of the *S*. *torvum* chromosome, and most of them were tandem repeats, which indicates that *NLR* has undergone genetic amplification during evolution, which is consistent with previous research results (Zhang et al., 2014). RNA-seq data also confirmed that all the three subgroups (TNL, CNL and PN) of 48 *NLR* genes were highly expressed in roots and fibrous roots (**Figure 6A** and **Supplementary Figure S9**), indicating that the *NLR* genes may be involved in the resistance to RKNs in *S*. *torvum*. Compared with eggplant in response to RKN infection (Zhang et al., 2021), three homologous *NLR* genes (*Sto0288260.1*, *Sto0201960.1* and *Sto0265490.1*) in *S*. *torvum* were significantly upregulated after RKN infection (**Figure 6B**).

**Figure 7:**
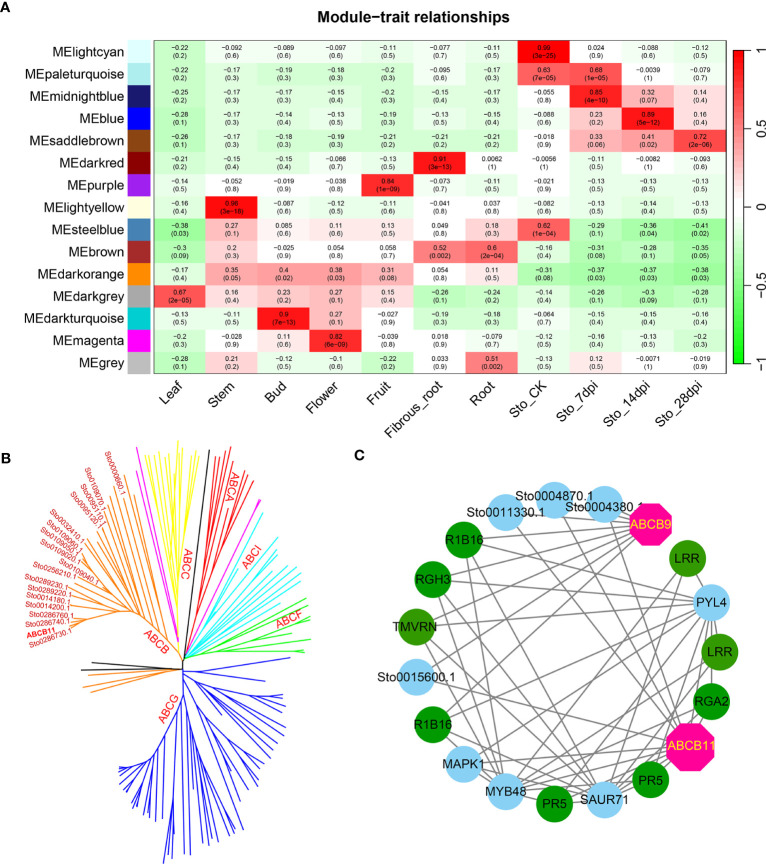
Co-expression network in different tissues and roots after RKN infection in *S*. *torvum* constructed by WGCNA. **(A)** The module-trait relationships showing the modules correlated with RKN infection. **(B)** Phylogenetic relationship of 139 ABC transporters in *S*. *torvum*. The unique expansion genes are labeled in red. **(C)** The subnetwork of the “saddlebrown” module with the hub genes in pink.

**Figure 8:**
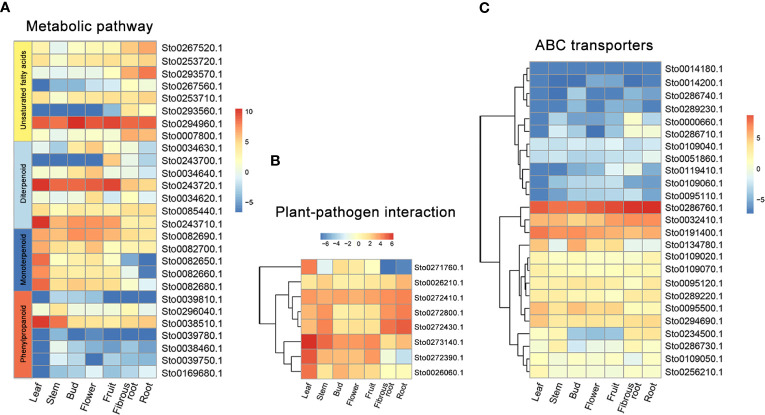
The expression patterns of specific expanded genes in different tissues of *S torvum*. **(A)** Genes involved in metabolic pathway. **(B)** Genes for plant-pathogen interaction. **(C)** ABC transporter genes.

**Figure 9:**
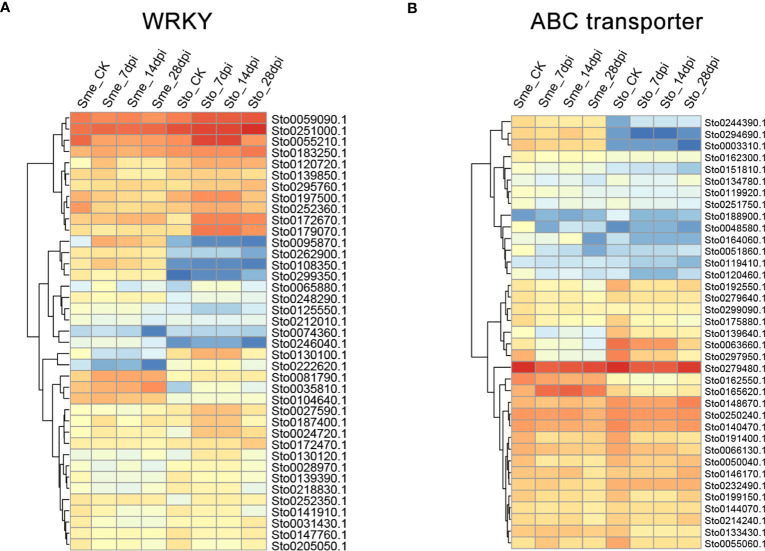
The expression patterns of WRKY **(A)** and ABC transporter genes **(B)** in *S*. *torvum* (Sto) and their homologous genes in *S*. *melongena* (Sme) in response to root-knot nematodes (RKNs).

A weighted gene co-expression network based on differentially expressed genes (DEGs) from RNA-seq data revealed that the genes in three modules (“blue”, “midnightblue” and “saddlebrown”) were significantly associated with RKN infection (**Figure 7A**). In our study, the *S*. *torvum* unique expansion genes contained 25 ABC transporter genes (ABCB subfamily), which may participate in RKN resistance (**Figure 7B** and **Supplementary Table S8**). In the “saddlebrown” module at 28 days post-inoculation (dpi), two ABCB genes, *ABCB9* (Sto0189000.1) and *ABCB11* (Sto0286710.1) were identified as the hub genes that interact with several disease resistance genes in response to RKN infection (**Figure 7C**). Particularly, *ABCB11* was one of the unique ABC transporter genes identified in *S*. *torvum*, implying its important role in RKN resistance (**Figures 7B, C**). The expression patterns of the expanded genes in different pathways were also showed that some of these genes were highly expressed in the root, including the 25 unique ABC transporter genes (**Figure 8**). In addition, the expression levels of the homologous genes in eggplant and *S*. *torvum* indicated WRKY and ABC gene families related to RKN resistance (**Figure 9**). Thus, some metabolite synthesis pathways and the unique *NLR* genes and ABC transporters may be involved in the resistance to RKNs in *S*. *torvum*.

### Chlorogenic acid is not involved in resistance to RKNs

**Figure 10:**
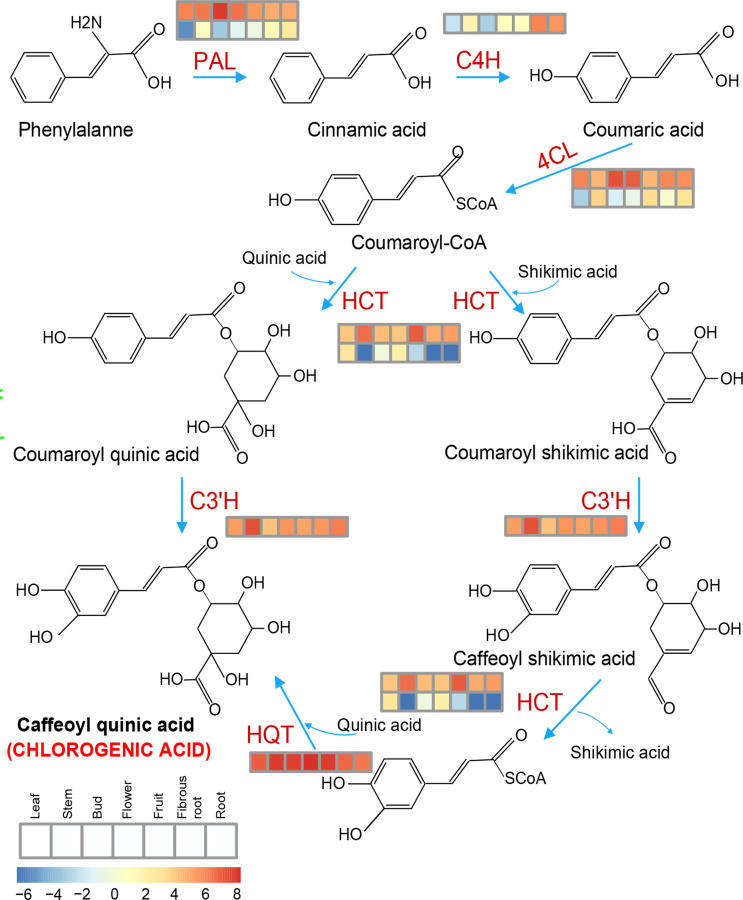
Key genes involved in the chlorogenic acid (CGA) synthesis pathway and their expression patterns in the different tissues of *S*. *torvum*. Expression levels with log2(FPKM).

Chlorogenic acid (CGA) is the phenolic metabolite of the phenylpropanoid pathway in several solanaceous plants, and plays an important role in plant defense (Plazas et al., 2013). In this study, some of the expanded genes in *S*. *torvum* were involved in phenylpropanoid biosynthesis, and genes associated with CGA biosynthesis were highly expressed in root tissues (**Figure 10**), which is consistent with other solanaceous plants, including eggplant and tomato (Li et al., 2021). However, the expression patterns of these genes were higher in eggplant than those in *S*. *torvum* after RKN infection (**Supplementary Figure S10** and **Table S12**). Thus, CGA might not be involved in the resistance of *S*. *torvum* to RKNs.

## Discussion

*Solanum torvum* (Turkey berry) is widely used as an important folk medicinal plant in tropical and subtropical countries and is also used as a rootstock in grafting for Solanaceae plants to improve the resistance to a soil-borne disease caused by RKNs (Gousset et al., 2005). RKN is one of the common pathogens for eggplant (*Solanum melongena*) and utilizes water and nutrients from the host eggplant, resulting in root and shoot growth retardation. Thus, Turkey berry can provide a valuable genetic resource for eggplant improvement (Sato et al., 2021). Herein, we present a chromosome-level assembly of genomic sequences for *S*. *torvum*, which will provide insights into understanding RKN resistance in Solanaceae plants.

The genome of *S*. *torvum* was assembled with the three different datasets of genomic DNA sequences and approximately 98.80% of the contigs were anchored into the 12 pseudo-chromosomes with an LAI of 10.47, indicating the high quality of the assembly (**Figure 2** and **Supplementary Table S3**). The annotated genes in *S*. *torvum* were comparable to those in other related species, indicating the high quality of prediction (**Supplementary Figure S4**). Genome collinearity analysis also revealed the conservation of genome sequences among the related species in *Solanum* genus. These results indicated that the assembled chromosome-level genome is highly complete and contiguous. Furthermore, comparison of the expanded and contracted gene families in *S. torvum* revealed that 368 gene families have been underwent expansion, including the *NLR* and ABC transporter gene families (**Figure 4A**). GO enrichment and KEGG pathway analysis of the *S*. *torvum* unique gene families suggested that they were involved in purine nucleotide metabolic process, chitin metabolic process and plant-pathogen interaction (Kyndt et al., 2012).

RKNs are one of the major pathogens of solanaceous crops and decrease root and shoot growth, resulting in considerable economic damage to crop production. In solanaceous crops, *R*-genes have been identified to confer resistance to RKNs and nearly all the *R*-genes reported were mapped to a collinear cluster of *NLR* genes within solanaceous genomes (Rutter et al., 2022). The tomato *Mi-1* gene that belongs to CNL type *NLR* gene, confers resistance to both RKNs and potato aphids (Vos et al., 1998). And *Mi1.2* gene that encode an NLR from tomato has been reported to confer resistance to *M. incognita* and *M. arenaria* (Milligan et al., 1998). In *Prunus*, a NLR gene *Ma* with TNL structure was reported for complete-spectrum resistance to Meloidogyne (Claverie et al., 2011). In our study, a total of 279 *NLR* genes along to the three subgroups (TNL, CNL and PN) were detected with 45 of TNL type, which is more number than eggplant (**Supplementary Figure S7**). Compared with the eggplant in response to RKN infection (Zhang et al., 2021), three homologous *NLR* genes (Sto0288260.1, Sto0201960.1 and Sto0265490.1) in *S*. *torvum* were significantly highly expressed after RKN infection (**Figure 6B**). And Sto0288260.1 is a CNL type NLR gene with significantly up-regulated in after RKN infection (**Figure 6B**), indicating the important role for RKN resistance. Furthermore, 48 *NLR* genes were highly expressed in roots and fibrous roots (**Figure 6**), indicating that the *NLR* genes play important roles in the resistance to RKNs in *S*. *torvum*. Therefore, further study of these *NLR* genes in *S*. *torvum* or eggplant might be elucidate their roles in resistance to RKNs.

In plants, ABC transporters have been reported to modulate resistance genes by improving the disease resistance of RKNs (Fujimoto et al., 2015). In this study, 25 *ABCB* genes were uniquely identified in *S*. *torvum*, indicating their roles in RKN resistance (**Figure 7** and **Supplementary Table S8**). Furthermore, the WGCNA revealed that two ABCB genes, *ABCB9* (Sto0189000.1) and *ABCB11* (Sto0286710.1) were identified as hub genes in response to RKN infection (**Figure 7C**). In rice, the ABC transporter OsPDR1 has been reported to regulate plant growth and pathogen resistance via affecting jasmonate biosynthesis (Zhang et al., 2020). And *FgABCC9* is required for fungicide resistance and pathogenicity toward wheat (Qi et al., 2018). In particular, knockdown of *ABC-C6* can inhibit egg hatching of *Meloidogyne incognita* in tomato (Cox et al., 2019). Thus, combining genome and transcriptome analyses revealed that the *ABCB11* gene which is a unique expansion gene of the *S*. *torvum*, plays an important role in the RKN resistance.

Chlorogenic acid (CGA) is a phenolic metabolite that is involved in plant defense (Plazas et al., 2013). In this study, the genes associated with CGA biosynthesis were highly expressed in root tissues, but the expression patterns of these genes were higher in eggplant than those in turkey berry after RKN infection, indicating that CGA might not be involved in resistance to RKNs in *S*. *torvum* (**Figure 10** and **Supplementary S12**). Thus, the induced resistance of *S*. *torvum* to RKNs could be caused via increasing secondary metabolite synthesis and the expression levels of unique *NLR* genes and ABC transporters.

## Conclusion

In this study, the high-quality genome assembly of *S*. *torvum* provides some new insights into the nematode resistance and lays a foundation for better elucidating the evolution and diversification of Solanaceae. Through gene prediction and annotation, 31,496 high-quality protein genes were annotated, and 27,719 (88.01%) of them were further detected by RNA-seq of seven tissues. A total of 368 gene families underwent expansion, including the *NLR* and ABC transporter gene families, and 707 gene families were contracted in the *S*. *torvum*. Integration of genomic and transcriptomic analyses revealed that secondary metabolite synthesis and the expression levels of unique *NLR* genes and ABC transporters may be caused to RKN resistance in *S*. *torvum*.

## Data availability statement

The datasets presented in this study can be found in online repositories. The names of the repository/repositories and accession number(s) can be found in the article/**Supplementary Material**.

## Author contributions

MZ, JH and JZ conceived and supervised the research. HZ, HC, MZ, XX, JT, SH, XC, HD and BW performed the experiments. HZ, JH, RZ, HD, and YW performed bioinformatics analysis. HZ, JH, MZ, ZH and JZ wrote and revise the manuscript. All authors contributed to the article and approved the submitted version.
