# Supplementary material for: The chromosome-scale reference genome and transcriptome analysis of Solanum torvum provides insights into resistance to root-knot nematodes
Source: Front Plant Sci. 2023 Jul 17;14:1210513. doi: 10.3389/fpls.2023.1210513 (PMC10390315; doi:10.3389/fpls.2023.1210513)
Supplement: Supplementary file 1 [file DataSheet_1.pdf]

Supplementary Figures

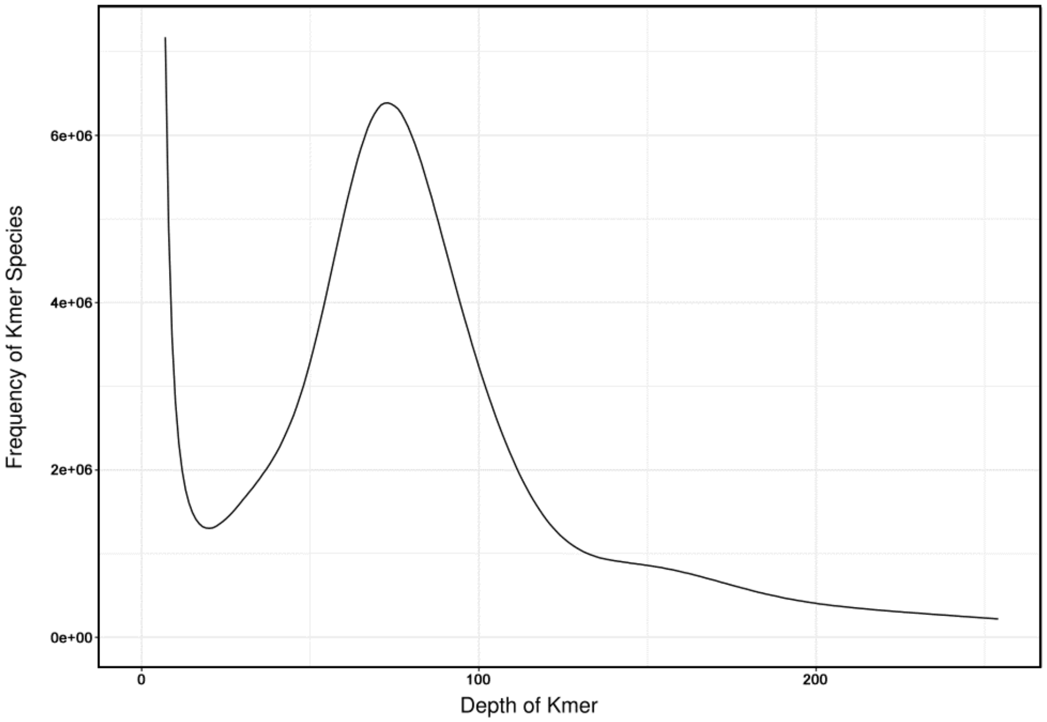

Supplementary Figure 1 K-mer analysis for estimating the genome feature of *Solanum torvum*.

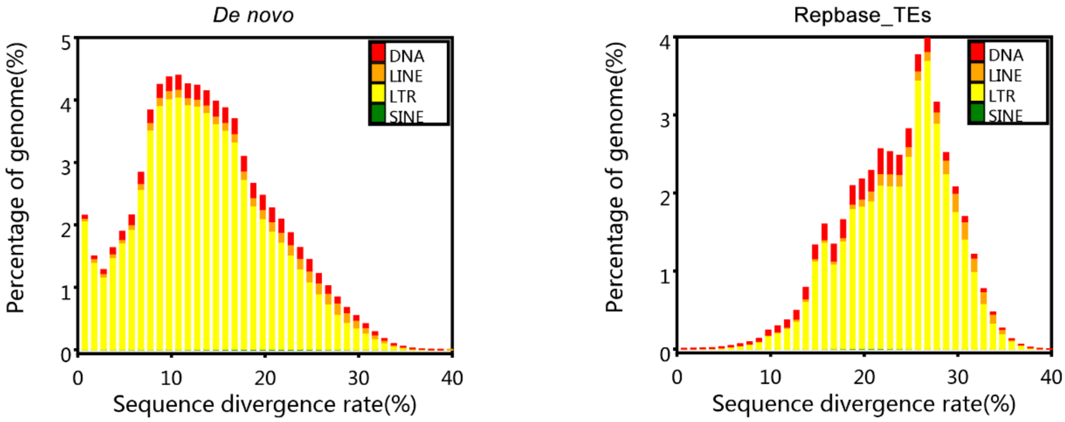

Supplementary Figure 2 Repetitive sequences predicted by *De novo* and based on RepBase in *Solanum torvum*.

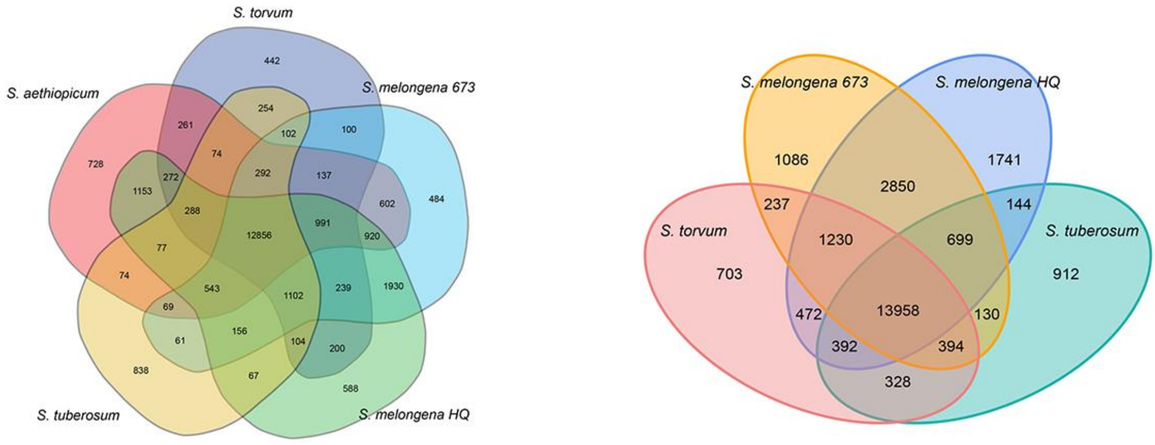

Supplementary Figure 3 Number of shared and unique gene orthologs in *Solanum* species.

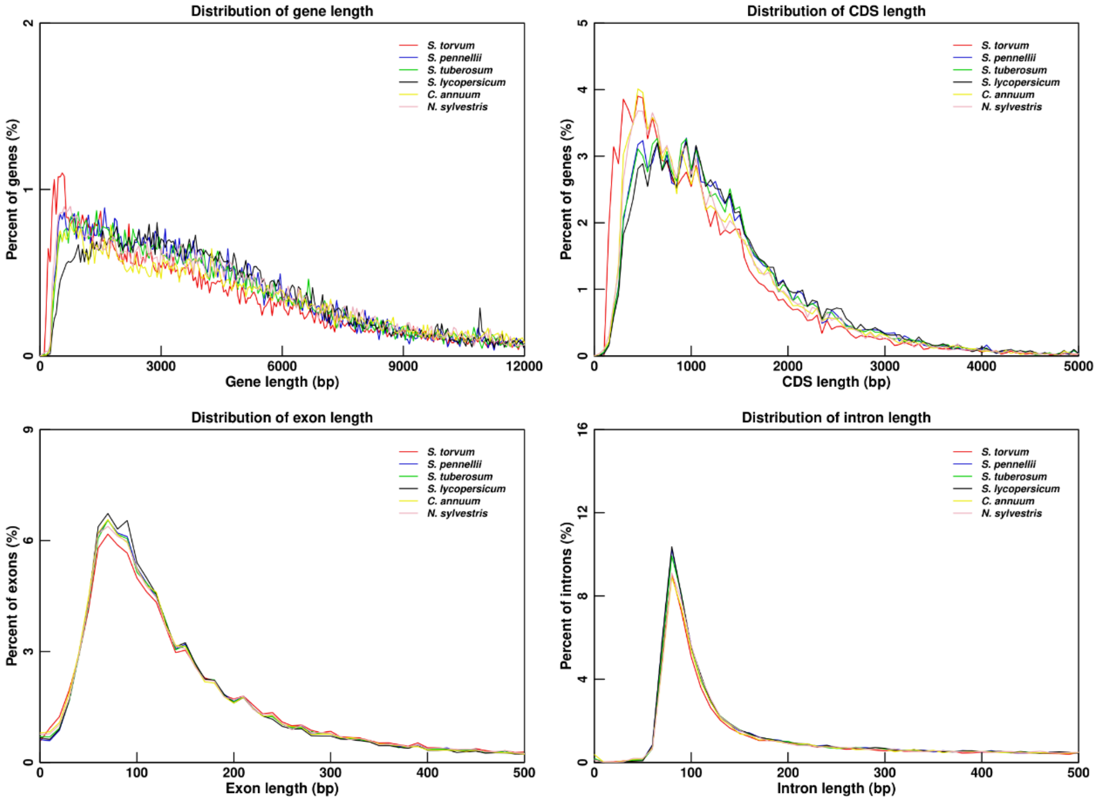

Supplementary Figure 4 Quality of gene annotation shown with gene length, CDS length, exon length and intron length in *Solanum torvum* genome, respectively.

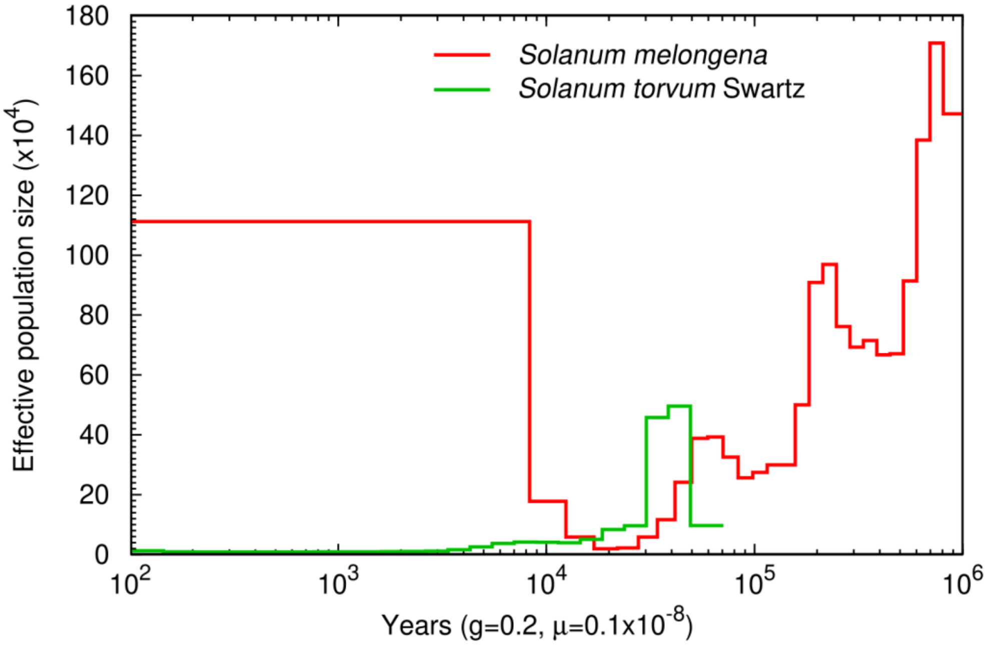

Supplementary Figure 5 Demographic N_e history of *Solanum melongena* and *Solanum torvum* inferred by PSMC analysis.

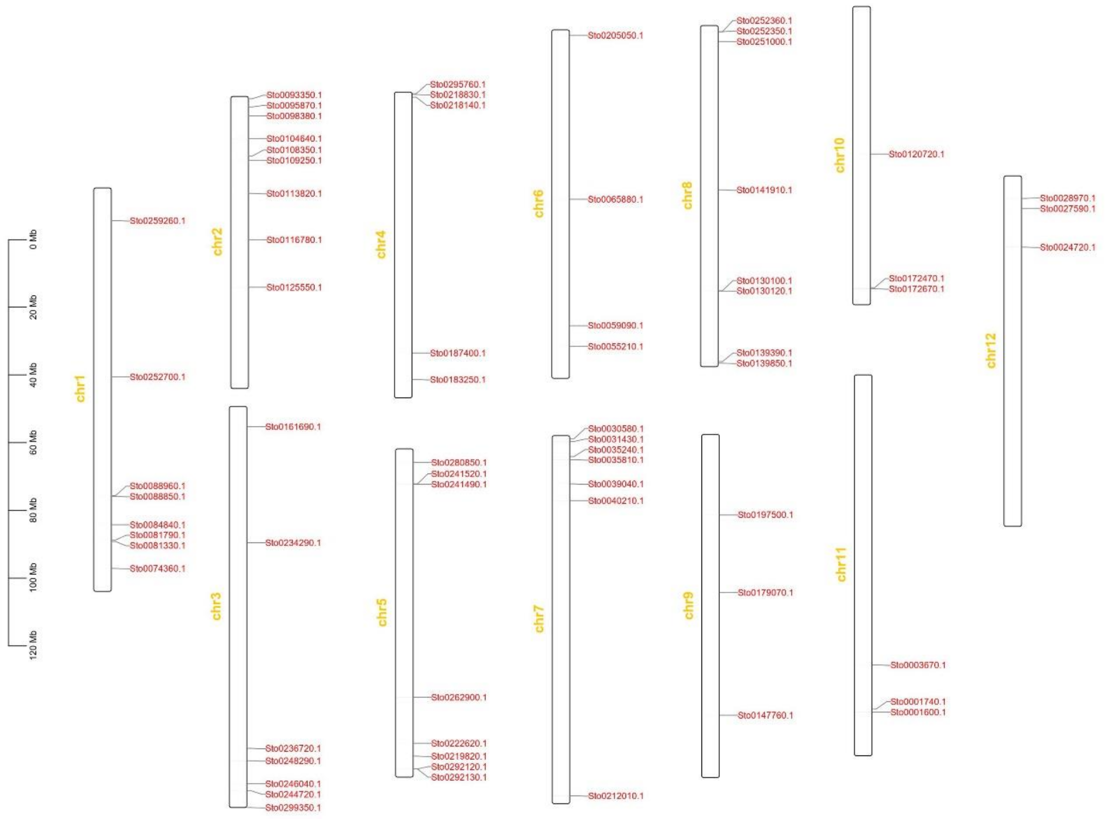

Supplementary Figure 6 The WRKY genes on *Solanum torvum* chromosomes.

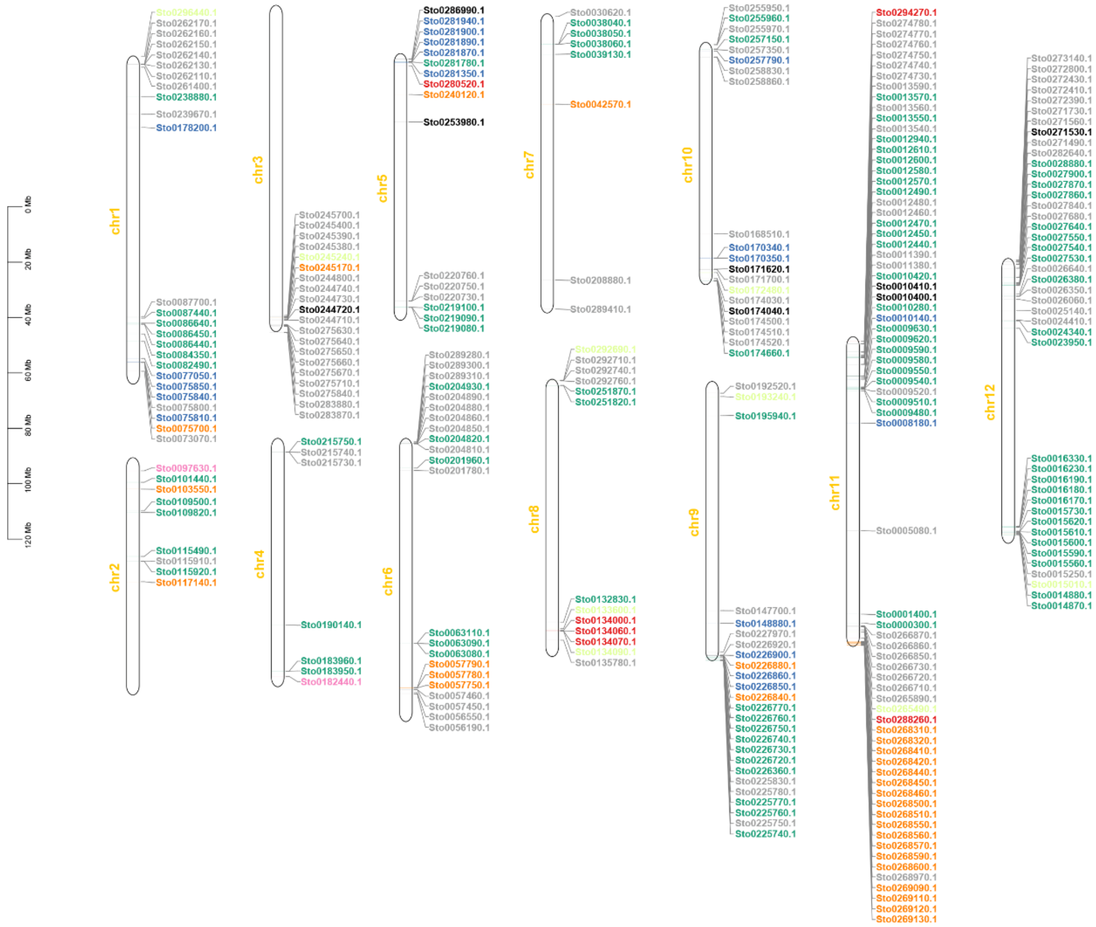

Supplementary Figure 7 The *NBS-LRR* genes on *Solanum torvum* chromosomes.

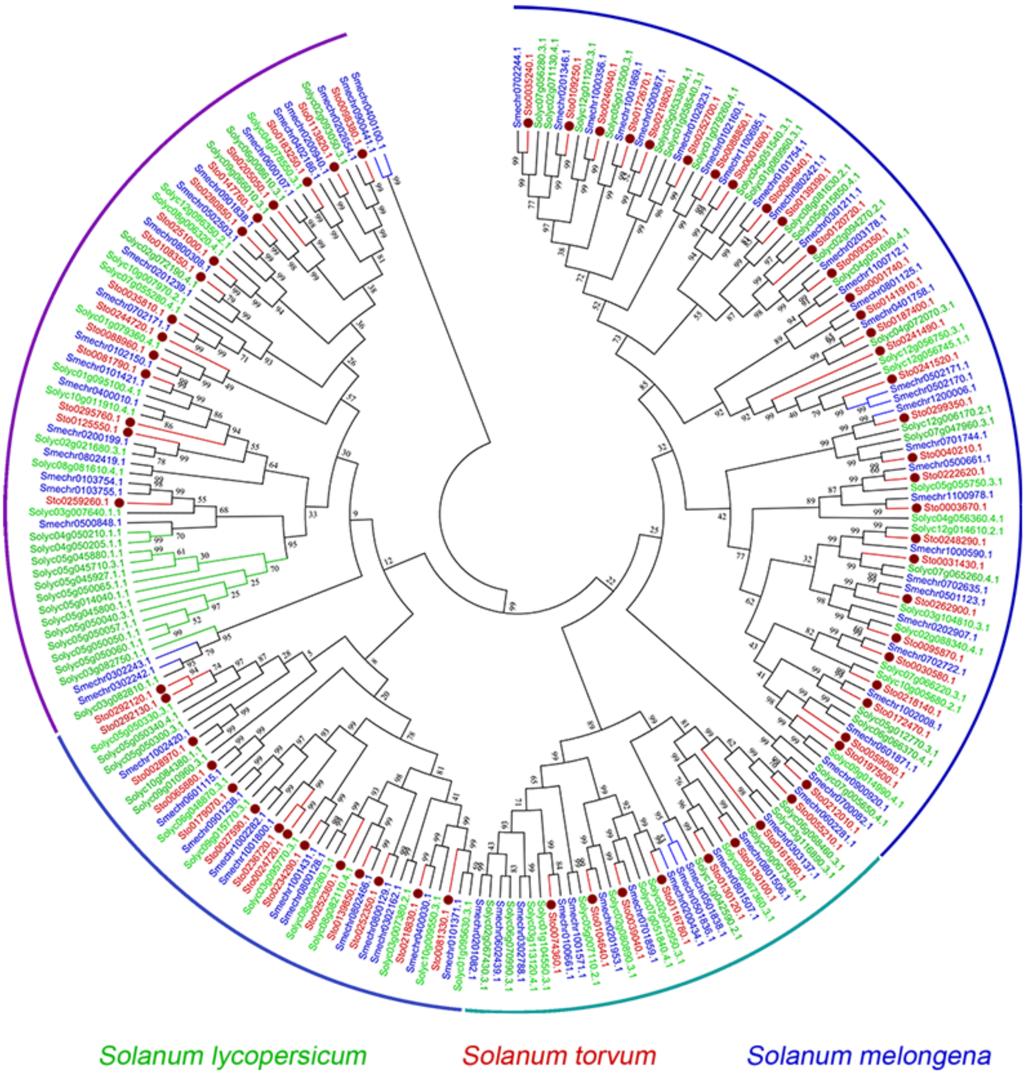

Supplementary Figure 8 Phylogenetic tree of the WRKYs in three species of *Solanaceae*.

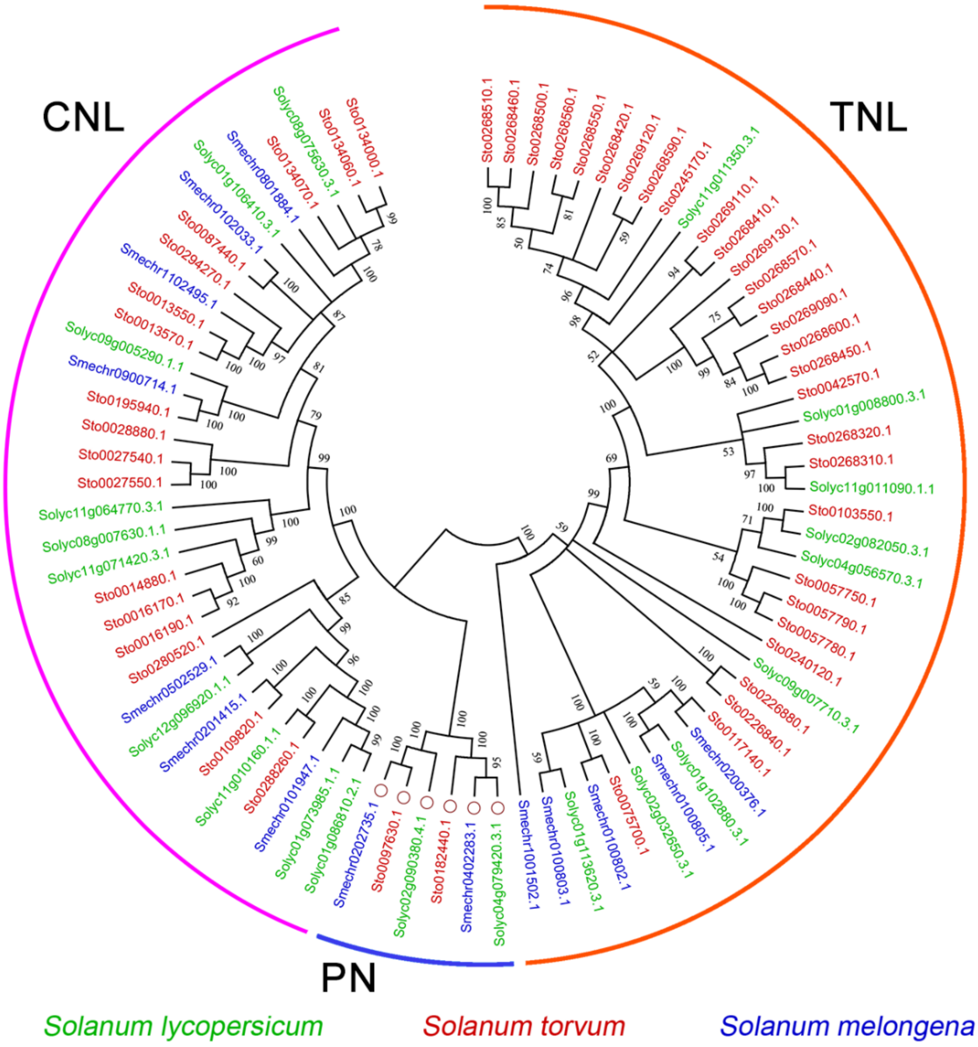

Supplementary Figure 9 Phylogenetic tree of the *NBS-LRR* genes in three species of *Solanaceae*.

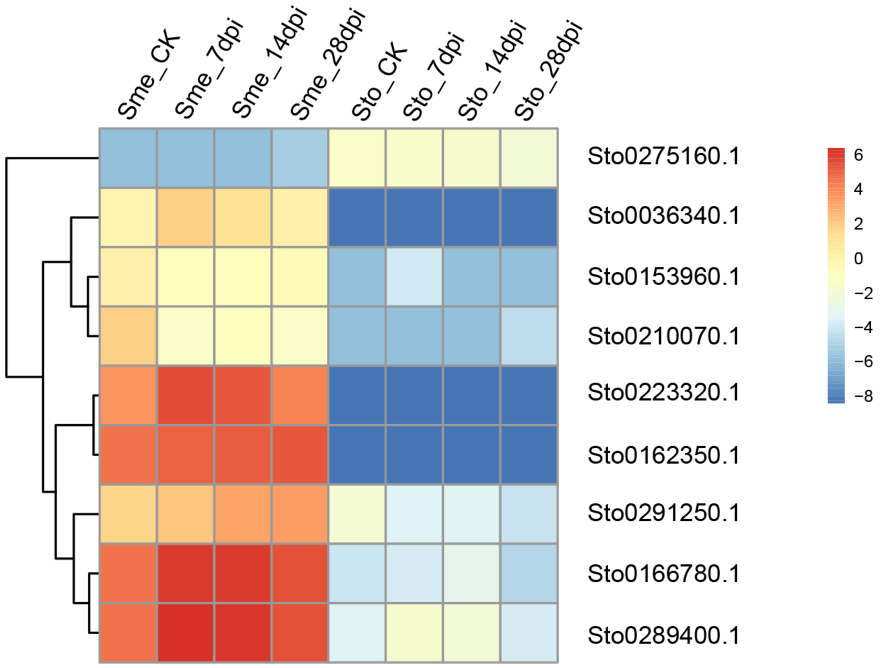

Supplementary Figure 10 The expression patterns of chlorogenic acid (CGA) related genes in *S. torvum* and *S. melongena* after RKN infection.
